# Dietary Intake and Its Contribution to Longitudinal Organophosphorus Pesticide Exposure in Urban/Suburban Children

**DOI:** 10.1289/ehp.10912

**Published:** 2008-01-15

**Authors:** Chensheng Lu, Dana B. Barr, Melanie A. Pearson, Lance A. Waller

**Affiliations:** 1 Department of Environmental and Occupational Health, Rollins School of Public Health, Emory University, Atlanta, Georgia, USA; 2 National Center for Environmental Health, Centers for Disease Control and Prevention, Atlanta, Georgia, USA; 3 Department of Biostatistics, Rollins School of Public Health, Emory University, Atlanta, Georgia, USA

**Keywords:** children, chlorpyrifos, dietary exposure, longitudinal pesticide exposure, malathion, organic diet, organophosphorus pesticides, urinary biomonitoring

## Abstract

**Background:**

The widespread use of organophosphorus (OP) pesticides has led to frequent exposure in adults and children. Because such exposure may cause adverse health effects, particularly in children, the sources and patterns of exposure need to be studied further.

**Objectives:**

We assessed young urban/suburban children’s longitudinal exposure to OP pesticides in the Children’s Pesticide Exposure Study (CPES) conducted in the greater Seattle, Washington, area, and used a novel study design that allowed us to determine the contribution of dietary intake to the overall OP pesticide exposure.

**Methods:**

Twenty-three children 3–11 years of age who consumed only conventional diets were recruited for this 1-year study conducted in 2003–2004. Children switched to organic diets for 5 consecutive days in the summer and fall sampling seasons. We measured specific urinary metabolites for malathion, chlorpyrifos, and other OP pesticides in urine samples collected twice daily for a period of 7, 12, or 15 consecutive days during each of the four seasons.

**Results:**

By substituting organic fresh fruits and vegetables for corresponding conventional food items, the median urinary metabolite concentrations were reduced to nondetected or close to non-detected levels for malathion and chlorpyrifos at the end of the 5-day organic diet intervention period in both summer and fall seasons. We also observed a seasonal effect on the OP urinary metabolite concentrations, and this seasonality corresponds to the consumption of fresh produce throughout the year.

**Conclusions:**

The findings from this study demonstrate that dietary intake of OP pesticides represents the major source of exposure in young children.

Organophosphorus (OP) pesticides, a group of cholinesterase-inhibiting insecticides, have been widely used both in residential and agricultural environments because of their broad spectrum of insecticidal activity and effectiveness. The widespread use of OP pesticides has led to frequent exposures in adult and child populations through multiple routes (Aprea et al. 2000; Hill et al. 1995; Lu et al. 2001). Infants and young children are often targeted for OP pesticide exposure assessment because of their susceptibility to possible neurologic and neurodevelopmental effects (Eskenazi et al. 2007; Whyatt et al. 2004). Although toxicologic and epidemiologic studies have demonstrated the association between acute and high levels of OP exposures and adverse health effects, the establishment of the relation between neurologic impairments and repeated low-level OP exposure that does not induce symptoms of acute poisoning in humans is far less concrete (Eskenazi et al. 2007; Rauh et al. 2006; Ruckart et al. 2004). The epidemiologic challenges inherent in exploring linkages between chronic OP pesticide exposures and adverse health outcomes may likely be attributable to the collection of exposure data that are not representative of true exposure. The frequently used one-time or spot measurement of urinary OP pesticide metabolites reveals only exposures that have occurred within a very short time frame and may provide very little evidence or insight regarding long-term or chronic exposures. Therefore, using spot biomarkers of OP pesticide exposure to examine the link between adverse health outcomes and cumulative OP pesticide exposure is an inadequate approach.

The objective of this article is to present the data assessing young urban/suburban children’s longitudinal exposure to OP pesticides in a group of young children participating in the Children’s Pesticide Exposure Study (CPES). The results from this study identify not only the predominant source of OP pesticide exposure but also the profile of exposures in children that are vital in formulating the strategies, for both regulatory policy and personal behavior change, in reducing children’s exposures to OP pesticides.

## Materials and Methods

### Study design

CPES was initially conducted in the suburban Seattle, Washington, area from summer 2003 through winter 2004 (CPES-WA) and was later repeated in Atlanta, Georgia, in 2006–2007 (CPES-GA) with the same study protocol. For both study locations, children participated in multiple-consecutive-day sampling periods in each of the four seasons. The data presented in this article are for the CPES-WA study only.

Twenty-three children 3–11 years of age were recruited from two local public elementary schools and a Montessori preschool in the Seattle area. Schools did not provide any assistance in recruiting subjects except for granting permission to send home with the children a letter and a fact sheet describing the study. Families that were interested in participating in this study then contacted the research group directly. A screening questionnaire was conducted over the phone to confirm eligibility, which included children who consumed exclusively conventional diets, spent most of their time in one residence, and had parents or caregivers willing to provide assistance in collecting specimen samples. Once a participant was enrolled, an in-house appointment was made to go over the study protocol, to train the parents/caregivers on how to collect specimen samples, and to obtain written consent from parents and older children or oral assent from younger children. A questionnaire was also administered during this appointment that asked about household pesticide use, to account for nondietary sources of pesticide exposure. The University of Washington Human Subjects Division (03-5899) approved the use of human subjects in this study, and the Emory University Internal Review Board (084-2005) approved continued analysis of this data set.

Each child committed to a 15- and a 12-consecutive-day sampling period in the summer (July–August) and fall (October–November) 2003, respectively, and a 7-consecutive-day sampling period in both the winter (January–February) and spring (April–May) 2004. In the summer and fall sampling periods, an organic diet substitution phase (from day 4 to day 8 for a total of 5 consecutive days) was incorporated into the study design to assess the contribution of daily pesticide exposures resulting from dietary intakes. We substituted most of the children’s conventional diets with organic items based on the grocery shopping lists provided by the parents or caregivers. Those food items included fresh fruits and vegetables, juices, processed fruits or vegetables (e.g., salsa), and some wheat- or corn-based items (e.g., pasta, cereal, popcorn, or chips). Children otherwise consumed their regular conventional diets for the remaining sampling days. No organic diet substitution was made during the winter and spring sampling periods. Selected organic food items, mostly fresh vegetables, fruits, and juices, were analyzed by a laboratory in Yakima, Washington, contracted by the U.S. Department of Agriculture (USDA) Pesticide Data Program (PDP) to confirm that the food items were indeed free of pesticides. No OP or other pesticides were detected in any of the organic food items analyzed.

### Specimen sample collection and analysis

For each sampling day, we collected two spot urine and saliva samples simultaneously, which included the child’s first morning void and the last void before bedtime. Saliva samples were collected using Salivette, a saliva collection device (Denovan et al. 2000). We also asked parents/caregivers to collect 24-hr duplicate food samples twice in the summer and once in the fall sampling period, as well as daily dietary consumption information throughout the four sampling periods. In the duplicate food sampling days (a total of 3 days), two additional spot urine and saliva samples, one after lunch and the other after dinner, were collected from the participants. We present and discuss only the urinary metabolite data in this article.

After collection, specimen samples were refrigerated or maintained on ice in a cooler before being collected by study personnel daily, and then were transported to the University of Washington research laboratory for measuring the total void volume and specific gravity for each of the urine samples. Urine samples were then aliquoted individually and stored at −20°C until the pesticide metabolite analysis was performed at the National Center for Environmental Health, Centers for Disease Control and Prevention (CDC), for metabolites of selected OP pesticides, pyrethroid insecticides, and herbicides (Olsson et al. 2004). In this article, we report results for OP pesticide urinary metabolites: malathion dicarboxylic acid (MDA) for malathion; 3,5,6-trichloro-2-pyridinol (TCPy) for chlorpyrifos; 2-isopropyl-4-methyl-6-hydroxypyrimidinol (IMPY) for diazinon; 3-chloro-4-methyl-7-hydroxy-coumarin (CMHC) for coumaphos; and 2-diethylamino-6-methyl-pyrimidin-4-ol (DEAMY) for methyl pirimiphos.

### Data analysis

The limits of detection (LOD) for the metabolites are constant for each urine sample analyzed and listed in Table 1. Concentrations of OP-specific metabolites were reported as three categories: detectable (> LOD), detectable but not quantifiable (< LOD), and nondetectable. For data analysis, we used reported concentrations for samples with > LOD and < LOD, whereas zero was assigned for nondetect samples. We calculated the daily volume-weighted average (DVWA) of OP pesticide metabolites (Equation 1) by averaging the metabolite concentrations in the morning sample (e.g., day 3) and the previous day’s bedtime sample (e.g., day 2), and then normalizing for the total volume of these two urine samples. This DVWA concentration was then considered as the estimate of the daily OP pesticide exposure (e.g., day 2 OP pesticide exposure). In cases where only one of the two urine samples was collected (< 2% of total samples collected), the metabolite concentration of the collected sample was used as the DVWA concentration. Urinary concentrations of OP metabolites were not adjusted by creatinine or specific gravity.


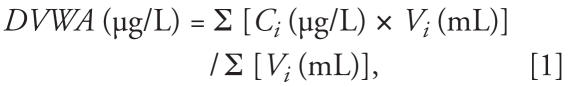


where *C_i_* is individual urinary concentration and *V_i_* is volume of the correspondent spot urine sample.

We used a linear mixed-effects model in SPSS 13 (SPSS Inc., Chicago, IL) with repeated urinary measurements for each participant to test the associations with season, age, and sex of children to determine whether these factors would influence their longitudinal OP pesticide metabolite concentration.

## Results

Twenty-three children were originally enrolled in the CPES-WA. Nine children were 3–5 years of age, and 14 children were 6–11 years of age. There were 13 males and 10 females, and they all lived in a suburban city near Seattle. Two children (both female, 3 and 8 years of age) dropped out of the study after the first session (summer period) because of difficulties encountered when collecting their specimen samples. In addition, two other children withdrew from the study, one (male, 5 years of age) after the fall and the other (male, 11 years of age) after the winter sampling period because of family reasons. CPES-WA ended with 19 children. A total of 724, 516, 260, and 257 spot urine samples were collected from the CPES-WA cohort over the summer, fall, winter, and spring sampling periods, respectively. More samples were missing in the first sampling period (summer), with 7% of total samples not collected. As families became familiar with the study protocol, the loss of samples decreased to 5% for the following fall and winter periods and 3% for the spring period.

**Figure 1:**
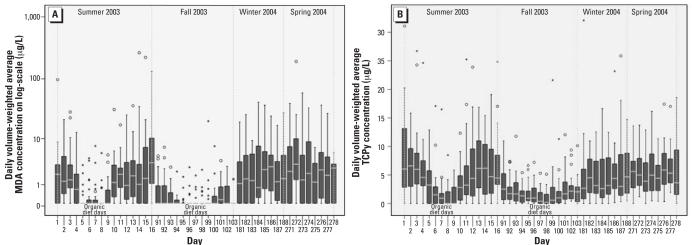
One-year exposure profile of DVWA of OP pesticide metabolite concentrations (μg/L) for CPES-WA children. Exposure data included urinary levels measured during the 5-day period in summer and fall 2003 when children consumed organic food items: (*A*) MDA; (*B*) TCPy. Days 5–9 and 95–99 were organic diet days. The horizontal lines in each plot represent 10th, 25th, 50th, 75th, and 90th percentiles, bottom to top. The concentration data on the *y*-axis is on the log-scale. The “o” and “*” symbols represent outliers and the extreme values, respectively.

The frequency of detection for different OP urinary metabolites varied (Table 1). TCPy, the specific urinary metabolite for chlorpyrifos, had the highest detection rate (91%) among the five OP metabolites that were targeted for analysis. MDA, the specific metabolite for malathion, had the second highest frequency of detection, followed by DEAMPY, CMHC, and IMPY (Table 1). The descriptive statistics for children’s longitudinal exposure to the five OP pesticides, expressed as the DVWA concentrations, are listed in Table 1. Because of the low detection frequency for DEAMPY, CMHC, and IMPY, only MDA and TCPy data were analyzed further (Table 2). Data for the urine samples collected during the organic diet days were included neither in the determination of the frequency of detection nor in the calculation of descriptive statistics. Those data were considered the outcomes from an intervention (organic diet substitution) protocol, and therefore did not represent children’s normal daily exposures to OP pesticides. However, when we examined the 1-year data including results from the days during which children consumed organic food items, a reduction in pesticide exposures brought about by substituting organic fresh fruits and vegetables for corresponding conventional food items was clearly observed for malathion (Figure 1A) and chlorpyrifos (Figure 1B) metabolites in both the summer and fall. Urinary concentrations of MDA and TCPy immediately began to decrease as soon as children switched to organic foods and continued to decrease during the 5-day organic diet intervention period. During both seasons, the elimination of malathion exposure among this cohort is most apparent; however, a decreasing trend is also visible for chlorpyrifos. As soon as children returned to their normal conventional diets, the DVWA of MDA and TCPy levels went back to the levels observed in the days before the introduction of organic diets.

**Figure 2:**
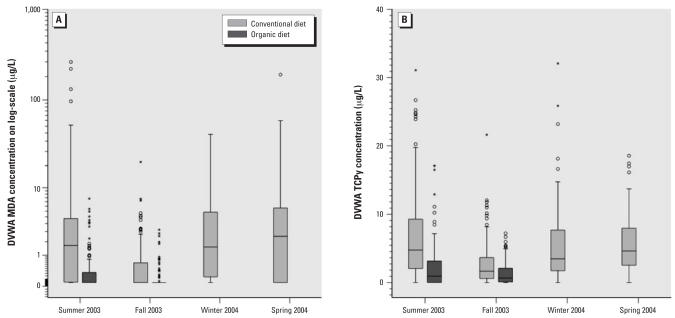
The distribution of the DVWA of OP pesticide metabolite concentrations (μg/L) in the CPES-WA children grouped by the consumption of conventional or organic food, and by different seasons: (*A*) MDA; (*B*) TCPy. The horizontal lines in each plot represent 10th, 25th, 50th, 75th, and 90th percentiles, bottom to top. The concentration data on the *y*-axis is on the log-scale. The “o” and “*” symbols represent outliers and the extreme values, respectively.

Figure 2 shows the comparisons of urinary concentrations of MDA and TCPy in the form of boxplots, illustrating overall differences when children consumed conventional instead of organic food and consumption during the four different seasons. In addition to the consumption of organic food, seasonality also seems to play a role in contributing to children’s exposures to OP pesticides. Both MDA and TCPy levels measured during the summer 2003 study session were higher than those in fall 2003, but were comparable to the levels measured in the winter and spring 2004 study sessions. The results from the linear mixed-effects model, in which we used the DVWA of MDA and TCPy levels as the repeated measurements within each subject, demonstrated that season was the only significant contributor to both MDA and TCPy levels in the urine (Table 3).

Considering the lack of residential use of OP pesticides among the families of CPES-WA children, consumption of conventional diets is likely to be the sole contributing factor to the seasonality effect of pesticide exposures. On the basis of the self-reported dietary survey, we counted the frequency of foods that were consumed daily by the children and grouped the foods into four categories: grains, fruits, juices, and vegetables. The results were then aggregated to produce the total consumption of each food category for each season, as well as consumption per child per day (Table 4). CPES-WA children consumed 89 different items of grains, fruits, juices, or vegetable types of food over the 12-month period. Among the 89 food items, apples, grapes, strawberries, apple and orange juices, lettuces (mixed salad), and peas are commonly consumed year round.

**Figure 3:**
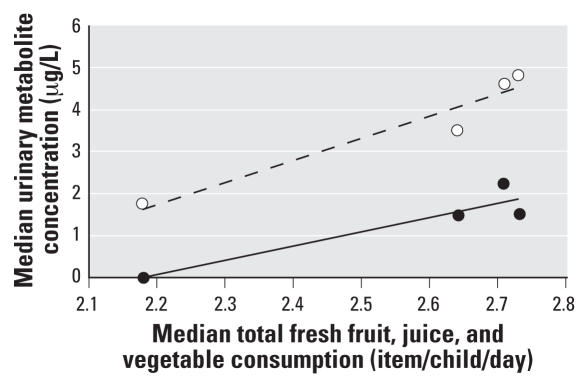
The correlation of the median DVWA of MDA and TCPy concentrations (μg/L) and the median consumption (per child per day) of total fresh fruits, juices, and vegetables for each of the four seasons. Solid line, MDA (*y* = 4.0963*x* − 10.405; *R* ^2^ = 0.5065). Dashed lines, TCPy (*y* = 5.2796*x* −9.8921; *R*^2^ = 0.9311).

Approximately 65% of food items that are commonly consumed by the CPES-WA children from 2003 to 2004 were included in the USDA PDP (USDA 2004) in which pesticide residues in selected food commodities are monitored by USDA yearly (Table 4). On average, each CPES-WA child consumed 3.05 items of fruits, juices, and vegetables combined per day in the summer season, which is higher, but not statistically higher, than consumption occurring in the other three seasons (Table 4). The CPES-WA children consumed the least amount of fresh produce (2.66 items per child per day) in the fall season, which coincided with the lowest DVWA concentrations of MDA and TCPy in the urine samples collected at the same period of time. Although the daily consumption of fresh produce (fruits, juices, and vegetables) is not correlated with each child’s DVWA urinary metabolite concentrations, the median urinary DVWA concentrations of both MDA and TCPy for the CPES-WA children as a group are significantly correlated with the median consumption of fresh produce for the four seasons (Figure 3) (Pearson, *p* = 0.04 for MDA; *p* < 0.01 TCPy). We did not ask the participants to record dairy products, meats, or seafood because OP pesticide residues are rarely found in these food items.

## Discussion

Few studies have attempted to quantitatively assess urban/suburban children’s exposure to pesticides in a longitudinal manner, although this group comprises the majority of children living in the United States. Most of the studies published in the literature either have targeted children living in agricultural environments or have used a cross-sectional design with spot sample collection (Barr et al. 2004; CDC 2007; Eskenazi et al. 1999; Fenske et al. 2000; Lu et al. 2001; Valcke et al. 2005; Whyatt et al. 2004), rendering their results incompatible for comparison to the CPES-WA results. The most apparent difference in the study design between CPES and other studies is that we employed a multiple consecutive-day sampling scheme in each of the four seasons with two successive (before bedtime and next morning void) urine and saliva sample collections each day. We implemented this study design based on the following goals: *a*) to better understand the long-term trend of pesticide exposures in children, *b*) to reduce the variability of measurements so the results would not dilute the true association of the outcomes of interest, and *c*) to consider the short biological half-lives of OP pesticides in the human body.

Although we successfully carried out this 1-year study (4 of 23 study participants at the end dropped out of the study), our study is not without limitations. First, this study was very resource-intensive, and with a limited research budget, we were not able to assemble a child cohort with a large number of study participants. Second, because of the demanding level of cooperation required from the parents/care-givers and their children to exactly follow the study protocol for a 12-month period, we screened interested families via phone interview to ensure their willingness to participate. Many of the families were deemed noneligible as a result of this process. Therefore, CPES-WA children likely do not mirror the general population of children in the United States, and the results from this study may not represent the pesticide exposures in other groups of children.

However, by incorporating an organic diet intervention protocol in the study design, the outcome of the exposure assessment allows us to segregate dietary exposures from overall exposures. The first two reports published from the CPES-WA based on the data obtained from the summer sampling period clearly indicated the validity of such a study design. We concluded that children are exclusively exposed to OP pesticides via their dietary intakes (Lu et al. 2006b), whereas dietary exposures to pyrethroid pesticides represent only a fraction of the total exposure (Lu et al. 2006a). The results were convincing because *a*) concentrations of urinary metabolites of OP pesticides decreased to below the level of analytic detection during the days when the organic diet intervention was in place, and *b*) no OP pesticides had been used by the families before or during this study. Results from the fall sampling period in which the organic diet intervention was repeated reinforces this conclusion. Children continued to be exposed to malathion (Figure 1A) and chlorpyrifos (Figure 1B) before and after the implementation of the organic food substitution, whereas during the 5-day organic consumption period, the exposures to these two OP pesticides were much lower and eventually reached nondetectable levels. The time it took to remove malathion and chlorpyrifos from the children’s bodies corresponds approximately to the reported biological half-lives of 8 and 36 hr for these two OP pesticides, respectively (Bouchard et al. 2003; Griffin et al. 1999).

We identified a seasonal effect of the urinary MDA and TCPy concentrations, and this seasonality is related to changes in the level of consumption of fresh produce among the CPES-WA children throughout the year. Children living in the suburban Seattle area have abundant access to many fresh fruits and vegetables during the summer, many of which contained malathion and/or chlorpyrifos residues detected by the USDA PDP in 2004 testing (USDA 2007), including apples, cantaloupe, grapes, lettuce, and strawberries. Other common fruits consumed by this cohort, but not included in the 2004 PDP testing for pesticide residues, include blueberries, peaches, plums, raspberries, and watermelon. The elevated summer OP pesticide exposures in CPES-WA children were likely linked to the consumption of those summer fruits. In contrast, when CPES-WA children consumed less fresh produce in the following fall season, their OP pesticide exposure correspondingly decreased. The significant correlation between the median consumption of fruits, juices, and vegetables and the median DVWA urinary concentrations among the CPES-WA children for each season supports this conclusion.

Although the explanation of lower OP pesticide exposures associated with less fresh produce consumption in the fall season is probable, other factors may explain the increasing exposures in the following winter and spring seasons, despite little change in fruit and vegetable consumption compared with the fall season. We observed that some of the fresh produce reported to have been consumed (Table 4) by the children during the winter and spring seasons, such as cantaloupe, grapes, lettuce, strawberries, tomatoes, and watermelon, may not have been grown in the United States. It is known from recent PDP results that the percent of imported fruits (such as grapes) and vegetables (such as tomatoes) testing positive for OP insecticides is often much higher than the percent of domestically grown produce, and that the levels of residues in imports are sometimes much higher than the residues in domestic produce. It is highly probable that most of the imported grapes, tomatoes, and other fresh produce entering the United States in any given year is consumed in the winter and spring season. Accordingly, we suspect that some of the food items consumed by the CPES-WA children during the winter and spring seasons contained higher OP pesticide residues than the matched items that they consumed in the summer. Evidence to support this hypothesis is contained in a report prepared by the U.S. Environmental Protection Agency (EPA) Office of Inspector General (U.S. EPA 2006), which showed a significant shift in residues and risk from domestically grown fruit and vegetables to imports since passage of the Food Quality Protection Act (1996). A dietary risk index (DRI) for specific foods tested by the PDP from 1994 to 2003 was developed that combines the frequency of residues, the level of residues found, and pesticide toxicity based on U.S. EPA registration data (U.S. EPA 2006). DRIs for the same 16 domestically grown and imported foods were calculated. The average domestic DRI fell from 225 in 1994 to 65 in 2003, whereas the average DRI for imports rose from 98 to 244 (U.S. EPA 2006).

The results from the CPES-WA study support a key conclusion stated in the National Research Council’s (NRC) *Pesticides in the Diets of Infants and Children* that “dietary intake of pesticides represents the major source of exposure for infants and children” (NRC 1993). Unfortunately, the other concern raised by the same NRC committee in recognizing that “the differences in dietary exposure to pesticide residues account for most of the differences in pesticide-related health risks that were found to exist between children and adults” (NRC 1993) remains unaddressed by this study. Despite the plentiful evidence of OP pesticides causing neurologic- and developmental-related adverse health outcomes in animals, evidence of such linkages is still relatively fragile in children (Eskenazi et al. 2007; Guillette et al. 1998; Rauh et al. 2006; Ruckart et al. 2004). In part, this can be attributed to the inability to accurately assess pesticide exposures and then convert those measurements to the matrices, in which the pharmacokinetics of pesticides in humans can be characterized and taken into account. The nature of the nonpersistent pesticides (short biological half-lives), transient and day-to-day fluctuation of exposures, significant within- and between-individual variability, and lack of human pharmacokinetic models for common OP pesticides all add different layers of complexity to this important research. The current prevailing cross-sectional studies designed to collect human specimen samples from individual children in the brief precursor time window is not sufficient to generate accurate exposure data that would allow us to investigate rigorously the association with adverse neurologic effects.

It is not our intention to advocate limiting consumption of fresh produce. In fact, it is vital for children to consume significantly more fresh fruits and vegetables than is commonly the case today, in the interest of sound nutrition and preventing troubling increases in childhood diseases, such as juvenile diabetes and obesity. Nor is our purpose to promote the consumption of organic food, although our data clearly demonstrate that foods grown organically contain far less pesticide residues. We are also not certain that the exposure levels for OP pesticides measured in this group of children whose diets consisted mainly of conventional food would cause any adverse health outcomes. However, a recent animal study has demonstrated that persistent cognitive impairment occurred in rats after chronic dietary exposure to chlorpyrifos accompanied by acute doses sufficient enough to induce symptoms of toxicity (Samsam et al. 2005). The implication of this animal study highlights the importance of assessing OP pesticide exposures longitudinally to increase the power of analyses searching for links between exposures and adverse health outcomes. In future work, we will analyze the urinary biomarker data using the pharmacokinetic approach to compare the absorbed pesticide doses with benchmark doses, such as the acute and chronic reference doses established by the U.S. EPA (2002). The outcomes from such analysis will provide important new insight into exposure and risk levels relative to those regarded by the U.S. EPA as consistent with the Food Quality Protection Act’s (1996) “reasonable certainty of no harm” standard.

In conclusion, we report the exposure profiles of two OP pesticides, malathion and chlorpyrifos, for a group of elementary school–age children participating in the CPES-WA study. This study is one of the first to quantitatively assess children’s longitudinal exposures to OP pesticides using repeated biological sample collection. The consistent finding of nondetectable OP urinary metabolites during the days when children switched to mostly organic diets reasserts the fact that OP pesticide exposure occurs predominantly through dietary intake. We observed a seasonal effect on OP urinary biomarker levels in this cohort, and this seasonality corresponds to the consumption of fresh produce among the children throughout the year. In addition, we have highlighted the possibility that this seasonal pattern in dietary pesticide exposure may be associated with whether the foods are either grown in the United States or imported. The findings from this study support the conclusion stated in the National Research Council’s 1993 report that dietary intake of pesticides represents the major source of exposure in infants and young children. However, further research efforts should be devoted to seek the links between OP pesticide exposures and the adverse health outcomes in children.

## Figures and Tables

**Table 1:** Descriptive statistics of DVWA concentrations (μg/L) of OP pesticide metabolites measured in CPES-WA children from summer 2003 to spring 2004.

	MDA	TCPy	IMPY	CMHC	DEAMPY
Mean (μg/L)	4.6	5.1	0.2	0.01	0.3
SD	17.1	5.0	1.2	0.02	1.3
No. of DVWA measurements^a^	702	701	677	702	675
Range (μg/L)	(0, 263)	(0, 32)	(0, 15)	(0, 0.5)	(0, 18)
LOD (μg/L)	0.3	0.2	0.7	0.2	0.2
Frequency of detection (%)	66	91	9	13	25
Percentile
5th	0	0	0	0	0
10th	0	0.2	0	0	0
25th	0	1.5	0	0	0
50th	1.6	3.7	0	0	0
75th	3.6	7.5	0	0	0.01
90th	8.9	11.3	0	0.02	0.5
95th	17.1	14.7	0.5	0.07	0.9

a These numbers do not include the measurements from the days (5 days) when subjects consumed organic diets.

**Table 2:** Descriptive statistics of seasonal DVWA concentrations of two organophosphorous pesticide metabolites, MDA and TCPy, measured in CPES-WA children from summer 2003 to spring 2004.

	MDA				TCPy			
	Summer	Fall	Winter	Spring	Summer	Fall	Winter	Spring
Mean (μg/L)	6.3	0.7	4.1	6.1	6.4	2.6	5.1	5.6
SD	24.6	1.9	6.7	17.6	5.9	3.1	4.9	4.0
No. of children	23	21	20	19	23	21	20	19
No. of DVWA measurements^*a*^	244	156	157	145	243	156	157	145
Range (μg/L)	(0–263)	(0–20)	(0–41)	(0–191)	(0–31)	(0–22)	(0–32)	(0–19)
Frequency of detection (%)	75	39	75	69	86	88	96	97
Percentile
5th	0	0	0	0	0	0	0.2	0.4
10th	0	0	0	0	0	0	0.6	1.3
25th	0	0	0.1	0	2.0	0.6	1.7	2.5
50th	1.5	0	1.5	2.2	4.8	1.7	3.5	4.6
75th	4.0	0.7	5.0	5.6	9.4	3.7	7.7	8.0
90th	9.8	1.6	9.5	12.1	15.0	6.0	10.7	11.4
95th	21.2	3.0	17.2	26.5	17.1	9.4	12.5	13.1

This table does not include the measurements from the days (5 days) when subjects consumed organic diets.

**Table 3:** Selected results of a linear mixed-effects model^a^ with the repeated measurement of DVWA urinary MDA and TCPy concentrations (μg/L) collected from CPES-WA children over a 12-month period.

		MDA		TCPy	
Source	Numerator df	Denominator df	*F*-value (*p* > *F* )	Denominator df	*F*-value (*p* > *F* )
Intercept	1	14.1	0.01 (0.9)	13.5	5.8 (0.03)
Season	3	685	4.2 (< 0.01)	680	21.9 (< 0.001)
Age	8	15	0.6 (0.7)	14.1	0.4 (0.9)
Sex	1	13.7	0.8 (0.4)	13.5	0.1 (0.7)

df, degrees of freedom.

a Model was developed in SPSS. Data obtained from the 5-day organic diet substitution period were not included.

**Table 4:** Consumption of fresh fruit, fruit juice, and vegetable items by CPES-WA children in 2003–2004.

	Summer 2003	Fall 2003	Winter 2004	Spring 2004
No. of participants	23	21	20	19
No. of sampling days	10^a^	7^a^	7	7
Fruits (total)	318 (1.38)	155 (1.05)	154 (1.10)	167 (1.26)
Apples	56 (0.24)	50 (0.34)	46 (0.33)	29 (0.22)
Blueberries^b^	28 (0.12)	0 (0)	1 (0.01)	2 (0.02)
Cantaloupe	25 (0.11)	9 (0.06)	6 (0.04)	12 (0.09)
Grapes	43 (0.19)	20 (0.14)	24 (0.17)	11 (0.08)
Oranges	11 (0.05)	20 (0.14)	21 (0.15)	16 (0.12)
Peaches	13 (0.06)	1 (0.01)	1 (0.01)	4 (0.02)
Pears	4 (0.02)	12 (0.08)	5 (0.04)	3 (0.02)
Plums^b^	10 (0.04)	2 (0.01)	2 (0.01)	0 (0)
Raspberries^b^	14 (0.06)	1 (0.01)	1 (0.01)	2 (0.02)
Strawberries	20 (0.09)	2 (0.01)	16 (0.11)	39 (0.29)
Watermelon^b^	26 (0.11)	2 (0.01)	2 (0.01)	11 (0.08)
Subtotal	250 (1.09)	119 (0.81)	125 (0.89)	129 (0.97)
Fruit juices (total)	194 (0.84)	90 (0.61)	114 (0.81)	101 (0.76)
Apple juice^b^	24 (0.10)	21 (0.14)	23 (0.16)	22 (0.17)
Lemonade^b^	30 (0.13)	2 (0.01)	4 (0.03)	5 (0.04)
Orange juice	63 (0.27)	32 (0.22)	40 (0.29)	24 (0.18)
Subtotal	117 (0.5)	55 (0.37)	67 (0.48)	51 (0.39)
Vegetables (total)	189 (0.82)	146 (0.99)	128 (0.91)	116 (0.87)
Cucumber	8 (0.03)	4 (0.03)	4 (0.03)	1 (0.01)
Lettuce	17 (0.07)	16 (0.11)	19 (0.14)	11 (0.08)
Mixed salad^b^	12 (0.05)	7 (0.05)	10 (0.07)	8 (0.06)
Peas^b^	14 (0.06)	10 (0.07)	5 (0.04)	12 (0.09)
Spinach	5 (0.02)	6 (0.04)	2 (0.01)	2 (0.02)
Tomato	9 (0.04)	5 (0.03)	8 (0.06)	4 (0.03)
Subtotal	65 (0.28)	48 (0.33)	48 (0.34)	38 (0.29)
Total consumption	701 (3.05)	391 (2.66)	396 (2.83)	384 (2.89)

Data are expressed as total consumption count (consumption count per child per day).

a Does not include the 5-day organic intervention period.

b Commodity that was not analyzed by USDA PDP for pesticide residues in 2004.
